# Autoimmune Diseases and Molecular Mimicry in Tuberculosis

**DOI:** 10.3390/biology13121083

**Published:** 2024-12-22

**Authors:** Leonid P. Churilov, Muslimbek G. Normatov, Hong Ling, Min Zhuang, Dmitry Kudlay, Anna Starshinova

**Affiliations:** 1 Department of Pathology and Laboratory of the Microangiopathic Mechanisms of Atherogenesis, Saint Petersburg State University, St. Petersburg 199034, Russia; l.churilov@spbu.ru (L.P.C.); muslimbek_normatov@mail.ru (M.G.N.); 2 Department of Microbiology and Immunology, Harbin Medical University, Harbin 150088, China; lingh@ems.hrbmu.edu.cn (H.L.); zhuangm@ems.hrbmu.edu.cn (M.Z.); 3 Department of Pharmacology, I.M. Sechenov First Moscow State Medical University, Moscow 119435, Russia; d624254@gmail.com; 4 Institute of Immunology FMBA of Russia, Moscow 115552, Russia; 5 Department of Pharmacognosy and Industrial Pharmacy, Faculty of Fundamental Medicine, Lomonosov Moscow State University, Moscow 119991, Russia; 6 Department of Mathematics and Computer Science, St. Petersburg State University, St. Petersburg 199034, Russia; 7 Almazov National Medical Research Centre, St. Petersburg 197341, Russia

**Keywords:** Tuberculosis, *M. tuberculosis*, molecular mimicry, autoantigens, autoimmunity, bioinformatics

## Abstract

Comorbidities in tuberculosis patients are increasing annually. Autoimmune pathology may influence the diagnosis and treatment of tuberculosis (TB). However, the molecular mimicry between *Mycobacterium tuberculosis* (Mtb) and human autoantigens is an important provocative factor in the development of autoimmunity on one hand. Mtb has already been widely discussed as a provocateur of autoimmunity in humans. The aim of this study was to determine whether molecular mimicry exists between Mtb antigens and human autoantigens previously demonstrated as targets of autoimmunity. Materials and Methods: We analyzed the level of antibodies in 19 patients with pulmonary tuberculosis. In all cases ELISA assays was used. Also, in parallel, we identified 29 similar pentapeptides between key Mtb antigens and human autoantigens. Bioinformatic methods were used in this study. All amino acid sequences of MT antigens and human autoantigens were obtained from the UniProt database, and similar epitopes between Mtb antigens and human autoantigens were identified using the original “Alignmentaj” program. The immunoreactivity of the shared pentapeptides in Mtb antigens was evaluated with use of the IEDB database. Results: The high level of antibodies to modified citrulinated vimentin (anti-MCV) was most frequently detected (57%) in comparison with other antibodies. Elevated levels of antibodies to C3 complement fragments (47%) and rheumatoid factors (21%) in the absence of any rheumatic or autoimmune diseases are noteworthy. Several of the shared pentapeptides belong to the immunoreactive epitopes of Mtb antigens. The bioinformatic data correlated with our earlier studies of the levels of corresponding autoantibodies in the sera of TB patients. Conclusion: Our findings on cross-reactivity and sequence similarity between the Mtb proteins and human autoantigens provide support for the role of antigen mimicry in TB-related autoimmunity.

## Simple Summary

Bioinformatic analysis of molecular mimicry is an important step in predicting the development of autoimmunity. The checking of microbial peptides for possible cross-reactivity can be useful for the development of safe vaccines and more effective immunotherapy. When analyzing autoantibodies in patients with pulmonary tuberculosis, we found a high level of antibodies to modified citrullinated vimentin (anti-MCV compared to other antibodies. Patients also had high levels of antibodies to C3 fragments of complement and rheumatoid factors in the absence of any rheumatic or autoimmune diseases. Some common pentapeptides are related to immunoreactive epitopes of Mtb antigens. We found that the bioinformatic data correlate with our earlier studies of the levels of relevant autoantibodies in the sera of tuberculosis patients. Our findings on cross-reactivity and sequence similarity between the Mtb proteins and human autoantigens provide support for the role of antigen mimicry in TB-related autoimmunity.

## 1. Introduction

*Mycobacterium tuberculosis* (Mtb) belongs to the family *Mycobacteriaceae*, and was discovered in 1882, by Robert Koch [1]. It causes tuberculosis (TB), which annually kills 3 million people across the world. According to the World Health Organization (WHO), nearly 10.6 million new cases of tuberculosis were detected in 2022, indicating an increase of 3.5% from the reported 10.3 million in 2021. After the COVID-19 pandemic, the incidence of tuberculosis increased by 3.9% from 2020 to 2022 [2,3]. WHO estimated that by 2023, about 44 million children aged 0–9 years and about 125 million adolescents aged 10–19 years would be infected with *M. tuberculosis complex* (Mtc). It is estimated that about 1.8 million children of different ages would suffer from TB. It should be noted that in 2021, 216 thousand children and adolescents died from it. According to current estimates, in 96% of pediatric TB cases have never received treatment [4].

A number of epidemiological studies suggest that infections may provoke the occurrence of autoimmune diseases [5,6,7]. Mtb is not an exclusion and can also facilitate the development of autoimmune diseases. Several incidences of distorted autoimmune reactivity regulation were documented in TB patients [3,8].

Pathological autoimmunity is characteristic of the attack of autoreactive lymphocytes and/or autoantibodies against intact self antigens. Autoimmune diseases are the third cause of death after cancer and heart disease worldwide [9]. An important mechanistic link of autoimmune diseases is the phenomenon of molecular mimicry [10].

Molecular mimicry occurs when microbial antigens have peptides similar to the epitopes of the host autoantigens, if the immune system uses these particular peptides for antigen presentation. In that case, anti-alien T-helpers can facilitate the activity of silent anti-self B-cells, or direct cross-reaction of anti-alien autoantibodies may occur [10,11]. Studying molecular mimicry or predicting similar peptides between microbial and host proteins is possible in silico using protein databases and computer programs.

The aim of this study was to investigate under TB conditions the molecular mimicry between Mtb antigens and some human autoantigens.

## 2. Materials and Methods

Due to the fact that the minimal immune determinant capable of inducing highly specific antibodies and determining specific immune interactions is formed by a pentapeptide 5 amino acid sequence [12,13], pentapeptides were used as sequence probes. We included data from 19 patients with bacterial excretion pulmonary tuberculosis to analyze autoantibody levels. The exclusion criteria included the presence of HIV infection, syphilis, neoplastic diseases, pregnant women, and decompensated diabetes mellitus. The level of antibodies to a mutated citrullinated vimentin (anti-MCV) was measured in the sera of 19 patients with pulmonary TB (Table 1).

### 2.1. Autoantibodies Determination

The patients positive for anti-MCV antibodies were evaluated for the presence of antibodies to cyclic citrullinated peptide (anti-CCP). Antibodies to anti-MCV were measured using ELISA (ORGENTEC, Mainz, Germany), and anti-CCP was measured with ELISA (Euroimmun, Lübeck, Germany). Additionally, the levels of the 17 most prevalent autoantibodies were determined. The following antibodies were analyzed: to thyroglobulin (-a-TG) and thyroperoxidase (-a-TPO), IgG antibodies against double-stranded DNA (-dsDNA), which were determined using ELISA (Euroimmune, Germany); antinuclear antibodies (-ANA), antibodies to neutrophil cytoplasmic antigens (-ANCA), to smooth muscle (-ASMA), antimitochondrial antibodies (-AMA), and antibodies to gastric parietal cells (-APCA), which were determined using indirect immunofluorescence (Euroimmune AG, Germany); the profile of antinuclear antibodies (SS-a,SS-B,Scl-70, Sm, CENP-B) was checked using immunoblot (Euroimmune, Germany); antibodies to beta2-glycoprotein (-b2GP) and to liver and kidney microsomes (-LKM), anticardiolipin antibodies IgM, IgG (-ACLA-G, ACLA-M), and antibodies to C1q complement factor (-a_C1q), which were determined using ELISA (ORGENTEC, Germany).

For the determination of seropositivity, the cutoffs proposed by the manufacturers were used.

For this study, 10 immunodominant antigens of Mtb were selected, based on the literature data [6 kDa early secretory antigenic target, ESAT-6-like protein EsxB, uncharacterized protein (Rv1507A), Isoniazid-induced protein IniB, Putative antitoxin VapB45, uncharacterized protein Rv1509, DNA-binding protein HupB, heat shock proteins Mtb-HsP60,h shock protein Mtb-HsP65, Mycobacterial catalase (mKatG)] and 8 human autoantigens were chosen based on the data of our previous studies [Vimentin (VIM), Insulin (INS), Thyroglobulin (TG), Myelin basic protein (MBP), Beta-1 adrenergic receptor (ADRB1), Apolipoprotein B-100 (APOB), Immunoglobulin heavy constant gamma 1 (IGHG1), and Immunoglobulin heavy constant gamma 3 (IGHG3)]—all of them were frequently targeted during highly spread autoimmune diseases. Earlier, we registered in TB patients elevated autoimmunity towards practically all these autoantigens [14,15,16].

All amino acid sequences of MT antigens and human autoantigens were obtained from the Uniprot database [17]. Bioinformatic methods were used to identify shared pentapeptides between Mtb antigens and human autoantigens, with the application of the original “Alignmentaj” program “https://github.com/muslimb/MyProekt1 (accessed on 15 September 2024)” registered by the Russian Federal Service of Intellectual Property (Certificate # 2023617186 of 29 March 2023). The algorithm of the program was described earlier [18].

To study the location of pentapeptides within 3D structures of human autoantigens, we used the PDB v3.1 and AlphaFold v3 [19,20,21] databases and the PyMol software v.3.1.3 [22].

IEDB databases [23] were used to evaluate the immunoreactivity of the pentapeptides from Mtb antigens.

### 2.2. Statistical Analysis

Statistical analysis was performed using GraphPad Prism 6 (Graph Pad Software, Boston, MA, USA), Statistica 10 (Statsoft, Hamburg, Germany) and MedCalc—version 18.2.1 (Ostend, Belgium) values. Flow cytometry data were analyzed using Kaluza software v2.3 (Beckman Coulter, Inc., Brea, CA, USA).

## 3. Results

According to the data presented in Table 2, the high level of antibodies to modified citrulinated vimentin (anti-MCV) was most frequently detected (57%) in comparison with the other autoantibodies checked.

Elevated levels of antibodies to the C3 complement (47%) and rheumatoid factor (21%) in the absence of any rheumatoid or autoimmune pathology are noteworthy.

In the next step, we analyzed molecular mimicry between Mtb antigens and human autoantigens. Bioinformatic analysis showed that Mtb antigens have 29 shared pentapeptides with human autoantigens selected on the basis of our previous studies and the literature data on autoimmunity in TB patients (Table 3).

**Figure 1:**
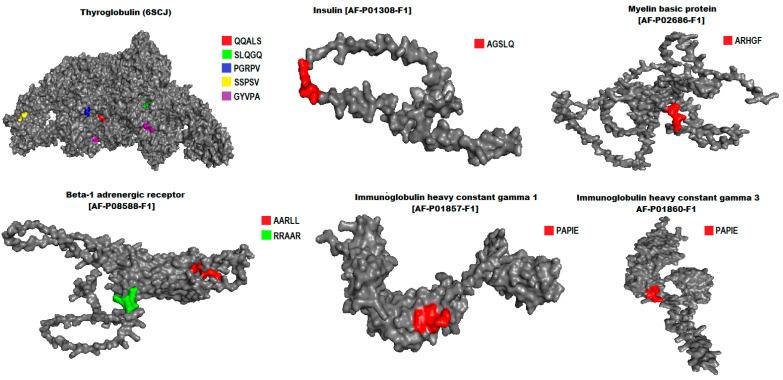
The location of pentapeptides in 3D structures of human autoantigens.

The locations of few similar pentapeptides in 3D structures of human autoantigens are shown in Figure 1.

Out of the 29 shared pentapeptides, 7 pentapeptides are located within the immunoreactive epitopes of Mtb for T and B cells according to the IEDB database (Table 4).

## 4. Discussion

The key to the diagnosis of autoimmune diseases is to determine the level of the most disease-specific autoantibodies. We have previously determined antibody levels and compared the results in patients with sarcoidosis and tuberculosis [16]. The mechanisms of the autoimmunity exaggeration in TB can be different. Among them, the adjuvant-like action of Mtb components, the aberrant expression of MHC Class II proteins on the somatic cells triggered by high levels of interferons, excessive cell death, and improper clearance of dead cell debris, with impaired autophagy, resulted in enhanced activation of antigen-presenting cells, etc. All the facets of this problem were discussed recently elsewhere [3].

However, one reason for the result obtained may be the mimicry of Mtb and self pentapeptides.

In total, 8 out of 29 common pentapeptides were revealed between Mtb immunodominant antigens and human thyroglobulin, which is the major target in autoimmune thyroid disease [24]. Two of the common thyroglobulin/Mtb pentapeptides are incorporated into immunoreactive Mtb epitopes for T- and B-immune cells. Interestingly, earlier we reported by immunoenzyme ELI-test elevated levels of autoantibodies to thyroglobulin (+59.1 ± 6.8% compared to average autoimmunoreactivity) in TB patients, but not in lung sarcoidosis [14,15]. The presence of anti-thyroglobulin autoantibodies in a 57-year-old BCG-vaccinated female with latent tuberculosis infection has also been reported [25].

We found six common pentapeptides between Mtb antigens and the human ApoB autoantigen. ApoB autoimmunity is associated with cardiovascular risk factors in humans [26]. The ability of TB to aggravate and accelerate atherosclerotic cardiovascular diseases has long been suspected and recently reviewed in detail elsewhere [27]. In addition, we determined similar pentapeptides between TB antigens and human INS, MBP, ADRB1, IGHG1, and IGHG3 autoantigens. Interestingly, our earlier wet lab studies demonstrated in TB increased levels of autoantibodies to all these autoantigens [14,15,16]. We found shared epitopes between the human VIM and Mtb proteins checked, while earlier we registered increased levels of autoimmunity against citrullinated VIM in TB patients [16].

Among all Mtb antigens studied, the heat shock proteins Mtb-HsP60 (P9WPE9, groEL1) had the greatest number of pentapeptides mimicking the epitopes of autoantigens.

Currently, several possible key mechanisms constituting the pathogenesis of autoimmune inflammation in tuberculosis are known, namely the following: adjuvant exposure to Mtb; molecular mimicry; impaired interaction between lymphocytes, dendritic cells, and the cytokine link of immunity; and a lack of proper immunoregulation caused by abnormal metabolism and individual immunogenetic features of the organism [3,28,29,30,31].

In many studies, autoantibodies to VIM and its modifications have been found in patients with sarcoidosis [32]. Sarcoidosis is known to be one of the granulomatous diseases with an autoimmune mechanistic component, where Mtb are listed among the trigger factors for inflammation [33]. A hypothesis was created about the molecular mimicry of VIM and mycobacterial proteins [34,35]. The heat shock proteins Mtb-HsP60, Mtb-HsP65, and catalase (mKatG) also are thought to be involved in cross-reactions with human peptides [36]. However, there have been no publications comparing the structure of VIM and mycobacterial proteins, hence the data obtained may open new perspectives for future studies.

A limitation of this study is related to its primarily focus on the analysis of protein sequence similarities. This study does not consider other factors that may contribute to autoimmune reactions, such as genetic predispositions. A finding of molecular mimicry between microbial antigens and human autoantigens in silico does not guarantee the development of autoimmunity, and the disease develops only if common epitopes are presented by antigen-presenting cells to lymphocytes. Nevertheless, several mimicking epitopes that our study revealed are surface-located within the 3D structure of proteins and listed as proven immunoreactive epitopes [37,38,39]. Currently, ESAT-6 and CFP-10 are actively used in the immunodiagnostics of tuberculosis infection in the structure of the main immunological tests, such as ELISPOT, Quantiferon TB testing, and Diaskintest [40].

The identified mimicry between ESAT-6-like protein EsxB (P9WNK5, esxB) and Insulin (P01308, INS)/Thyroglobulin (P01266, TG) proteins define the need for further studies on the impacts of widely accepted immunological tests on patients with endocrine pathology. The difficulties in detecting the latent TB infection in patients with diabetes mellitus are well known [41,42]. It is possible that the presence of mimicry between the presented proteins is one of the reasons for these hardships, and thus it requires the performance of other tests for early diagnosis of TB infection in this risk group.

Autoimmune thyroiditis is related to autoimmunity against thyroid proteins with alternative splicing, creating their isoforms dependent of environmental and other factors. This phenomenon may cause the appearance of neo-antigenic variations in thyroglobulin [43]. In context of TB, it is worth mentioning that some anti-TB medicines may alter the biosynthesis of thyroglobulin, thus facilitating autoimmunity towards its modification [44].

## 5. Conclusions

Although the development of autoimmunity after tuberculosis has long been reported, molecular mimicry between tuberculosis antigens and human autoantigens has rarely been studied in silico. Bioinformatic analysis of molecular mimicry is an important step in predicting the development of autoimmunity. The checking of microbial peptides for possible cross-reactivity can be useful for the development of safe vaccines and more effective immunotherapy. The findings on cross-reactivity and sequence similarity between the Mtb proteins and human autoantigens correspond to earlier wet lab studies [14,15,16,32] and provide support for the role of mimicry in TB-related autoimmunity.

The clinical correlations of such studies have been discussed in a recent comprehensive literature review, postulating that “Elucidating the role of molecular mimicry in human autoimmunity could have important clinical implications”. [42]

In view of broad molecular mimicry between Mtb and human autoantigens, the global burden of TB may contribute to the global prevalence of autoimmune diseases. Thus, the biological phenomenon of molecular mimicry predicted almost 120 years ago by Russian zoologist Konstantin S. Mereschkowski (1855–1921) as an element of his symbiogenesis theory of eukaryotic cell origin [45] seems to be translated into the medicine of today.

Taking such findings into account is essential in order to personalize medical care to TB patients prone to autoimmune diseases or with autoimmune comorbidities, because the immunosuppressive therapy of autoimmunopathies makes this cohort of patients less resistant to TB [46].

Identifying in TB patients the presence and concentration dynamics of autoantibodies against the antigens listed above may be recommended for follow-up analysis to validate the in silico findings, a task that has been achieved just partially so far.

## Figures and Tables

**Table 1:** Demographic characteristics of patients with pulmonary tuberculosis.

Characteristics of Patients	Pulmonary Tuberculosis, n (%) (n = 19)
Gender	
Men	9 (47.3)
Women	10 (52.7)
Age	32.5 (±4.5) years
Bacterial excretion	19(100.0)
MDR-Mtb	5 (26.3)
Smoking	5 (26.3)
Allergy	3 (15.7)
Diabetes mellitus	0
HIV infection	0
Hepatities	0
Chest CT changes, n (%):	
Disseminative changes in the lungs	5 (26.2)
Focus on the lungs	4 (21.1)
Infiltrative changes	10 (52.7)
Complaints, n (%):	
Fever	6 (31.8)
Cough	9 (47.3)
Dyspnea	3 (15.7)

**Table 2:** High autoantibody levels in tuberculosis patients.

Antibodies	Reference Values	Patient with Tuberculosis (n = 19)	
		abs. (M; 95%Cl)	High Antibody Levels (n/%)
Level of anti-MCV	>19.5 U/mL	23.4 19.17–27.61	11 (57.9)
Level of C3 component of complement, C3, g/L,	N 0.75–1.65 g/L	1.16; 0.98–2.81	9 (47.4)
Rheumatoid factor, RF, ME/mL,	N < 20	27.4; 20–144	4 (21.1)
Antibodies to beta2-glycoprotein 1, B2GP total, RU/mL,	N < 20	20.3; 0.2–156.81	3 (15.7)
Antibodies to Saccharomyces cerevisiae Ig, GASCA GRU/mL,	N < 20	13.5; 0.64–86.29	3 (15.8)
Level of C4 component of complement, C4, g/L,	N 0.13–0.54 g/L	0.40; 0.17–0.6	3 (15.8)
Antibodies to thyroglobulin TG, IU/mL,	N < 100	161.3; 2.15–2.423	2 (10.5)
Anti-TPO antibodies, IU/mL	n < 50	47.5; 4.92–364.42	2 (10.5)
Antibodies to cyclic citrulline peptide, ACCP, IU/mL	n < 5	1.54; 0.01–15.6	1 (5.3)
Cardiolipin antibodies, CL M, U/mL	N < 10	2.3; 0.95–11.7	1 (5.2)
Antibodies to HER-2 cell antigens	1:1280–40,000-high	168.8; 160–320	1 (5.4)
DNA antibodies, IU/mL,	N < 25	3.13; 0.1–56.12	1 (5.2)
Smooth muscle antigens (SMAs)	<1:40	40	2 (3.6)

**Table 3:** Molecular mimicry between MT antigens and human autoantigens. (PDB ID, protein name).

Autoantigens//Mtb Antigens	6 kDa Early Secretory Antigenic Target (P9WNK7, esxA)	ESAT-6-Like Protein EsxB (P9WNK5, esxB)	Uncha-Racterized Protein (L7N6B6, Rv1507A)	Isoniazid-Induced Protein IniB (P9WJ97, iniB)	Putative Antitoxin VapB45 (O53464, vapB45)	Uncha-Racterized Protein Rv1509 (P9WLW3, Rv1509)	DNA-Binding Protein HupB (P9WMK7, hupB)	Heat Shock Proteins Mtb-HsP60 (P9WPE9, groEL1)	Heat Shock Protein Mtb-HsP65 (P9WPE7, groEL2)	Mycobacterial Catalase (mKatG) (P9WIE5, katG)	Total
Vimentin (P08670, VIM)	-	-	-	-	-	-	-	-	REKLQ, EKLQE	DNASL, FGGPG, EEIQE	5
Insulin (P01308, INS)	-	AGSLQ	-	-	-	-	-	LLPLL	LLPLL	-	3
Thyroglobulin (P01266, TG)	-	QQALS, SLQGQ	PGRPV, SSPSV	-	-	GYVPA	-	SLPDL, VTGGQ, EVLGS	-	-	8
Myelin basic protein (P02686, MBP)	-	-	-	ARHGF	-	-	-	-	-	-	1
Beta-1 adrenergic receptor (P08588, ADRB1)	-	-	AARLL	-	-	-	RRAAR	LLIVA	AATAA	-	4
Apolipoprotein B-100 (P04114, APOB)	-	-	SASVH	AGLAS	ELELR	-	-	ALIKG, ALTEL	-	LTVSQ	6
Immunoglobulin heavy constant gamma 1 (P01857, IGHG1)	-	-	-	PAPIE	-	-	-	-	-		1
Immunoglobulin heavy constant gamma 3 (P01860, IGHG3)	-	-	-	PAPIE	-	-	-	-	-		1
Total	0	3	4	4	1	1	1	7	4	4	**29**

**Table 4:** Immunoreactive epitopes of MT for T and B cells. Pentapeptides are shown with **bold** script.

Epitope ID	T Cell Epitopes	Epitope ID	B Cell Epitopes
194400	t**AGSLQ**gqw	33831	ktqidqvest**AGSLQ**
653350	**AGSLQ**gqwrgaag	52689	qvest**AGSLQ**gqwrg
127789	**QQALS**sqmgf	655	adeeq**QQALS**sqmqf
851940	eeq**QQALS**sqmgf	70686	vqysradeeq**QQALS**
194400	tag**SLQGQ**w	1681	ag**SLQGQ**wrgaagta
37668	**LLPLL**ekvigagkpl	52689	qvestag**SLQGQ**wrg
13839	eqi**AATAA**isagdqs	103744	yd**REKLQ**erlaklag
		103744	ydr**EKLQE**rlaklag
		103605	skvstvkd**LLPLL**ek
		51040	qi**AATAA**isa

## Data Availability

Availability of data and materials. All source data are in this article; if you need clarifications, or need additional information, you can write to muslimbek_normatov@mail.ru.
